# Reimagining open science for global health: Epistemic power and the pursuit of health equity

**DOI:** 10.1371/journal.pgph.0005300

**Published:** 2025-11-06

**Authors:** Alice Rangel Teixeira, Ricardo Kleinlein, Nai Lee Kalema, Gift Osei Agyemang, Charles Senteio, Leo Anthony Celi, Seth Agyemang, Meskerem Aleka Kebede

**Affiliations:** 1 Department of Philosophy, Universitat Autònoma de Barcelona, Barcelona, Spain; 2 Department of Anesthesiology, Perioperative and Pain Medicine, Brigham and Women’s Hospital, Harvard Medical School, Boston, Massachusetts, United States of America; 3 Institute for Innovation and Public Purpose, University College London, London, United Kingdom; 4 Department of Medical Laboratory Science, University of Energy and Natural Resources, Sunyani, Ghana; 5 Department of Library and Information Science, Rutgers School of Communication and Information, New Brunswick, New Jersey, United States of America; 6 Laboratory for Computational Physiology, Massachusetts Institute of Technology, Cambridge, Massachusetts, United States of America; 7 Division of Pulmonary, Critical Care and Sleep Medicine, Beth Israel Deaconess Medical Center, Boston, Massachusetts, United States of America; 8 Department of Biostatistics, Harvard T.H. Chan School of Public Health, Boston, Massachusetts, United States of America; 9 Directorate of Surgery, Komfo Anokye Teaching Hospital, Kumasi, Ghana; 10 Global Surgery Policy Unit, LSE Health, London School of Economics and Political Science, London, United Kingdom; Institute of Tropical Medicine: Instituut voor Tropische Geneeskunde, BELGIUM

## Abstract

This paper critically examines how current forms of Open Science (OS) fall short of advancing health equity in global health. While OS is promoted as a public good, promising transparency, efficiency, and inclusive, current practices often reproduce rather than dismantle entrenched inequities. Data-sharing infrastructures and open-access policies, largely shaped by high-income countries, frequently extract from but fail to empower low- and middle-income countries. Appeals to transparency likewise overlook deeper asymmetries in whose knowledge is recognized, whose labor is valued, and whose communities benefit from scientific advances. We argue that in the context of global health, OS must be re-imagined not as a technical reform but as a political and epistemic project oriented toward health equity. Drawing on feminist, de-colonial, and Black feminist scholarship, we show how global health knowledge has been structured through histories of exclusion that continue to shape categories, standards, and priorities. Building on these critiques, the paper advances five guiding commitments for re-imagining OS: epistemic plurality, redistribution of resources, accountability to marginalized communities, co-creation and participatory governance, and reflexivity and care. Rather than a prescriptive model, these commitments offer enabling conditions for a more equitable and pluralistic science, reclaiming imagination as a vital resource for collective transformation.

## Introduction

Open Science (OS) has gained global prominence as a strategy for improving transparency, efficiency, and collaboration in research [1,2]. Policy documents, funding agendas, and institutional practices present OS as a universal good: a way to democratize knowledge, accelerate discovery, and ensure accountability across the research process [3–5]. Within global health, OS has been promoted as a pathway to equity by widening access to knowledge and making data and publications more readily available.

Yet these narratives often obscure how OS operates within uneven global and epistemic hierarchies. By privileging technical openness over structural transformation, OS risks reinforcing rather than dismantling inequalities [6–11]. Data-sharing infrastructures designed in high-income contexts may extract knowledge from, but fail to empower, low- and middle-income countries [9,12,13]. Appeals to transparency often overlook deeper asymmetries of power that determine whose knowledge counts as credible, whose labor is recognized, and whose communities benefit from scientific advances [14–17].

Existing scholarship has begun to highlight these tensions, pointing to the digital divide, barriers to meaningful participation, and the persistence of epistemic exclusion despite open-access policies [6,7,12–14,18]. While these critiques expose important practical shortcomings, they often leave the underlying epistemological foundations of OS intact. Rarely is OS questioned as more than a technical reform, and the possibility of re-imagining it as an epistemic project remains under-explored.

This paper addresses that gap by advancing a set of guiding commitments for the radical re-imagination of OS in global health. Drawing from feminist, de-colonial, and Black feminist scholarship, we argue that OS must move beyond a narrow focus on technical openness toward confronting epistemic authority and its role in reproducing inequities. Our contribution is twofold: theoretically, we reconceptualize OS in global health as an epistemic project oriented toward health equity; and practically, we identify five commitments: epistemic plurality, redistribution of resources, accountability to marginalized communities, co-creation and participatory governance, and reflexivity and care; that can guide equity-centered scientific practice. In doing so, we expand OS from a project of efficiency to one that contributes to the pursuit of health equity through epistemic transformation.

## The rise of Open Science

Over the past few decades, there has been a growing movement aimed at transforming scientific practice to better align with the demands and opportunities of the digital era, as well as to enhance the societal impact of science. Key milestones in this movement include the 1999 UNESCO/ICSU Declaration on Science and the Use of Scientific Knowledge, the Science Agenda – Framework for Action [3]; the 2002 Budapest Open Access Initiative [5]; the 2003 Bethesda Statement on Open Access Publishing [19]; and the 2003 Berlin Declaration on Open Access to Knowledge in the Sciences and Humanities [4]. These initiatives represent a broader trend toward making scientific knowledge more accessible and equitable, shaping debates in global health, where access to data, publications, and research infrastructures is frequently presented as a condition for achieving health equity [10,11].

The equitable, Open Access (OA) to research findings, methodologies, and the inclusion of diverse stakeholders in the scientific conversation has been shown to advance research endeavors significantly. Notable examples include the INSPIRE initiative, which has catalyzed research in high-energy physics [2], and the rapid growth of computer sciences, particularly through the Free Software movement [20], which has been one of the greatest drivers of the digital revolution and widespread adoption of computer-based technologies. In global health, similar arguments link OS to the acceleration of research on communicable diseases and to the integration of underrepresented regions into scientific collaboration [21]. More recently, the potential of Artificial Intelligence to advance health has increased attention to Open Science (OS) as a path to overcome some limitations of the technology, such as the need for huge volume of data, data complexity and concern about the privacy of patient information [10].

In line with these trends, UNESCO’ s 2021 Recommendation on Open Science advocates for a global framework to promote transparency, accessibility, and inclusivity in scientific research and data sharing [22]. According to the report, Science, Technology and Innovation (STI) is critical in providing solutions for global challenges, as evidenced by the global COVID-19 crisis, which underscored the importance of freely sharing scientific knowledge, data, and methodologies to respond to future global health and other crises. For global health specifically, UNESCO highlights the transformative potential of OS to close STI and digital gaps, particularly in developing regions, and to accelerate the achievement of the Sustainable Development Goals (SDGs) related to health.

Still, as we argue in this paper, the mere articulation of these principles is necessary but not sufficient to ensure their effective implementation. For instance, the COVID-19 response depended on OS, allowing the rapid sharing of genomic sequence, epidemiological data, and treatment protocols that allowed for an expedited drive for vaccine development and a well-coordinated global response [23]. However, the peak of COVID-19 pandemic was heralded by disparities in vaccine distribution, largely due to existing structural injustices that an equitable implementation of OS could avert. Structural inequalities, differences in resources, and varying levels of digital infrastructure across global regions create significant barriers to achieving truly Open Science (OS). Beyond pandemics, global health research continues to reflect unequal power relations, with priorities often shaped around the needs and perspectives of the Global North rather than those of the communities most affected [16]. These examples suggest that achieving health equity requires not only wider access but also a rethinking of the epistemic foundations of OS itself, including whose knowledge is valued, who sets research priorities, and how benefits are distributed.

## The Open Science paradox

Despite the increasing prevalence of OS in scientific literature, its definition remains elusive and is often described as a “vague mix of ideals” [6]. Among the many formulations, the one most frequently cited comes from UNESCO, which defines OS as “an inclusive construct that combines various movements and practices aiming to make multilingual scientific knowledge openly available, accessible and reusable for everyone” [22], Other frameworks broaden this further: the Berlin Declaration emphasizes free and sustainable access to scholarly work, while the OECD highlights transparency, reproducibility, and participation in the research process. Taken together, these definitions suggest that OS is not only about accessibility but also about fostering collaboration, transparency, and inclusivity across the research life cycle. Within global health specifically, OS is invoked in three main ways 1) as a tool for transparency and reproducibility (for example, ensuring clinical trials or epidemiological data can be checked for accuracy and fraud) [21]; 2) as a mechanism for efficiency and acceleration (sharing data to speed vaccine or drug development, particularly visible during the COVID-19 pandemic) [24]; and 3) as a framework rhetorically tied to equity, where access to knowledge is framed by influential global health actors, such as the WHO and leading journals, as a pathway to reduce health disparities [11,25].

OS is therefore presented as a corrective to systemic problems in contemporary science [7]. These include the continued dominance of publication practices by institutions in the Global North [7], the over-reliance on data from Western, Educated, Industrialized, Rich, and Democratic (WEIRD) countries [26–28]. and the historical exclusion of marginalized groups such as women [29] and racial minorities [28], from clinical trials, producing biased data that neglects the health needs of diverse populations. Moreover, collaborations between researchers in the Global North and Global South sometimes reproduce colonial dynamics through so-called “helicopter research,” in which studies are conducted in low- and middle-income countries (LMICs) with minimal involvement of local scientists, resulting in outcomes that often fail to meet local needs [8,9,30].

Although OS is premised on overcoming such inequities, the reality is more complex. While it aspires to create equitable and universal access to knowledge, barriers such as high article processing charges (APCs) in open-access journals disproportionately affect researchers from LMICs [12,13], This contributes to lower authorship rates from the Global South in OA publications [31], mirroring historic inequities in knowledge production. At the same time, financial pressures can push underfunded researchers toward predatory journals, which compromise quality and credibility [7,32]. These obstacles affect entire disciplines, with Humanities and Social Sciences often facing greater difficulties than STEM fields due to limited funding opportunities. Given that OA articles typically achieve higher citation rates, and researcher performance is evaluated through publication and citation counts, visibility remains skewed toward better-resourced groups and topics [33,34]. Compounding these inequities, editorial boards of leading scientific journals are still dominated by Western scholars [32], who often select peer reviewers from their own networks [35] disproportionately favoring men from North America, Europe, and Asia [36–38].

Some scholars, like Syed [39], argue that this lack of diversity stems from the fact that OS has not developed into a social-structural movement. By this, Syed refers to a collective reorganization of the social and institutional conditions under which science is produced; one that actively redistributes power, authority, and resources in knowledge production. Instead, OS has largely been implemented as a technical or policy fix, without addressing entrenched inequities. Consequently, current OS practices reproduce existing injustices in knowledge dissemination and fail to consider the social power dynamics that shape scientific participation. Critiques from scholars such as Bezuidenhout [11] and Ross-Hellauer [7,40]underscore this point, showing that equity in OS often remains rhetorical, invoked by dominant institutions but rarely translated into structural change. This limited scope can be seen in contexts such as the COVID-19 pandemic, where OS enabled rapid sharing of research but did not guarantee equitable access to the resulting benefits. If OS is meant to address “complex and interconnected environmental, social and economic challenges for the people and the planet” [22], how can it do so when its most visible applications, such as during COVID-19, reproduced rather than reduced inequities?

## Global health and epistemic power

If OS is meant to address “complex and interconnected” global challenges, its limitations during COVID-19 suggest that the problem lies less in the model itself and more in how it is taken up within fields already shaped by inequities in knowledge production, as is the case of global health. Conditions disproportionately affecting the Global South, such as neglected tropical diseases (NTDs); or the rising burden of non-communicable diseases (NCDs), for example, diabetes, hypertension, and dyslipidemia, continue to receive limited scientific attention and funding, despite their massive public health impact [41,42]. Diseases such as leprosy, leishmaniasis, and lymphatic remain underrepresented in mainstream research agendas, even though they affect over 1.6 billion people globally [42]. These disparities are not only a matter of political will or funding, but also reflect underlying epistemic hierarchies — including whose knowledge counts, which health problems are seen as urgent, and how evidence is defined and validated.

In principle, OS could help correct these imbalances by enabling knowledge and data sharing, fostering equitable collaborations between Global North and South, and amplifying locally produced research in LMICs. Such approaches could integrate culturally relevant perspectives into disease prevention, management, and policy frameworks [15]. Yet in practice, local knowledge rarely informs global policy. Global health research is still shaped predominantly by Northern authors and institutions, reinforcing a *foreign gaze* [43] that determines priorities, frames findings, and marginalizes Southern voices. These epistemic asymmetries sustain structural inequities in what counts as legitimate knowledge and who gets to produce it. Consequently, instead of fulfilling its promise, OS risks becoming a tool that perpetuates extractive practices in global health, facilitating the widespread sharing of data from LMICs without ensuring appropriate agency or benefits afforded to local researchers and populations. The COVID-19 pandemic provides a stark example of how openly shared knowledge has failed to equitably benefit populations across the globe.

Beyond resource distribution problems that lead to the under-representation of the Global South in many areas of health care, issues of epistemic domination by specific regions, institutions, and social groups have shaped global health knowledge. Historically, little attention has been given to questions of power asymmetries in knowledge production [44]. As Shiffman [16] argues, global health is structured not only by material inequalities but also by epistemological power: the capacity to create meaning through categories such as burden of disease, cost-effectiveness, or the ethical principles that should guide health policy. Structural biases condition the design of health policies, while more diffuse epistemological power, such as the authority to define what counts as legitimate knowledge or who qualifies as an expert, shape global research agendas and standards of evidence [17]. Through these forms of epistemic authority, actors from the Global North disproportionately determine global health priorities and the terms of care, with significant consequences for populations in the Global South. This makes clear that health equity also depends on addressing epistemological power, an argument long emphasized by feminist and de-colonial scholars.

Feminist and de-colonial scholars show that these epistemological hierarchies are not accidental, but embedded in broader structures of power that determine which bodies are visible, which needs are prioritized, and which ways of knowing are considered legitimate. Black feminists such as Dorothy Roberts [45] and Angela Davis [46] expose how the exclusion of Black women’s experiences from dominant feminist and medical frameworks reflects deeper hierarchies of race, class, and gender. Health disparities — such as the much higher rates of maternal mortality among Black women in countries like Brazil [47] and the United States [48], or the persistent inequities faced by Indigenous peoples worldwide, alongside the marginalization of Indigenous knowledge [49] — are not only consequences of structural racism but also expressions of a colonial legacy of epistemic power that continues to shape health systems and research priorities. Concepts like *epistemic injustice* [50] and *epistemicide* [51] describe how certain populations are systematically excluded not only from care but from the categories and evidence bases that define legitimate knowledge.

De-colonial feminism builds on these critiques by showing how colonialism continues to organize knowledge itself. Scholars such as María Lugones [52] and Kimberlé Crenshaw [53] have argued that these axes of oppression [53], such as gender, class and race, must be understood as co-constituted through *colonialism of power* [54] that persists in modern institutions, including science and medicine. This critique reveals how global health continues to privilege Global North-centered approaches while marginalizing community-based, Indigenous, and experiential knowledge. In response, de-colonial feminist frameworks call for epistemologies grounded in the lived realities of historically oppressed populations; highlighting, for example, the contributions of Indigenous healing systems globally [49,55,56]. By naming how health knowledge is entangled with histories of domination, these frameworks urge a transformation not just of access, but of the terms through which knowledge in global health is defined and legitimized, something that technical initiatives like Open Science, on their own, cannot achieve.

Taken together, these critiques show that the challenges facing global health are not simply about widening access or including more voices in existing models, but about confronting the epistemological foundations on which those models rest. This means that Open Science cannot be treated as a neutral set of practices or technical protocols, it must be reimagined as a political and epistemic project. As Joan Tronto [57] points out, existing structures of knowledge are not neutral containers to which excluded groups can simply be added. They have been built through exclusions, such as of women, racialized populations, linguistic and cultural minorities, so that the very shape of science reflects those histories of exclusions. This is why the inequalities exposed by feminist and decolonial critiques cannot be addressed through incremental reforms or technical guidelines. What is needed is a shift in how we conceptualize science itself, its purposes, its epistemic boundaries, and its relationships to power.

Drawing on both theoretical insights and practical experiences from feminist and decolonial movements, we argue that such reimagining requires what Iris Marion Young [58] identifies in her critique of distributive justice: a radical shift in the imagination, through which we reconsider the very norms and assumptions that define justice itself. In a similar way, addressing inequities in global health requires rethinking the norms, ideals, and assumptions that structure how knowledge, human lives, and health systems are understood. Health equity, in this light, depends on rethinking not only what knowledge is and who produces it, but also the purposes it is made to serve. Radical imagination provides the collective capacity needed for this rethinking.

Radical imagination emerges from the analysis of social movements that have enacted transformation under conditions of structural violence and marginalization [59]. Developed most prominently in Max Haiven’s work, the concept is tied to the relationship between social movements and academic research. Rather than treating research as an external, neutral activity, Haiven argues that it participates in the very processes through which movements reproduce themselves and sustain struggle. Researchers, then, are not only observers but also responsible for enlivening and convoking the radical imagination that sustains social transformation [59]. Applied to open science, this is not a methodological exercise but a political and epistemic necessity. Radical imagination names the collective capacity to envision futures foreclosed by dominant systems, to sustain resistance in the present through practices of solidarity and care, and to reinterpret both past and present in ways that expand the possibilities of action. It is not an individual possession or abstract ideal, but a shared capacity that undergirds political and epistemic transformation [59]. Applied to global health, radical imagination is best understood as a collective effort to rethink how OS can move beyond technical openness to genuinely advance health equity.

From our review of feminist, decolonial, and Black feminist epistemologies, together with Iris Marion Young’s account of imagination as a tool for rethinking justice and Max Haiven’s theorization of radical imagination, we identify five central commitments for a radical re-imagination of OS. These commitments were developed inductively, by tracing recurring themes that emerge when these theoretical traditions engage with questions of knowledge production, power, and equity in health contexts. They are not a prescriptive checklist but mutually reinforcing conditions that can guide both research practice and institutional policy.:

1. **Recognition of epistemic plurality** – valuing diverse forms of knowledge, including Indigenous, community-based, and practice-based epistemologies, as equally legitimate contributions to scientific inquiry [49,56,60].
2. **Redistribution of resources and infrastructures** – addressing structural inequities by ensuring equitable access to funding, infrastructures, and platforms necessary for meaningful participation in OS [61,62].
3. **Accountability to marginalized communities** – embedding ethical responsibility by ensuring that openness does not exacerbate extraction, exploitation, or exclusion [19,25].
4. **Co-creation and participatory governance** – democratizing decision-making processes in OS by involving diverse stakeholders in agenda-setting, design, and oversight [61].
5. **Reflexivity and care in knowledge practices** –recognizing that imagination is situated: what different communities envision as the goals and practices of OS will vary. Scientific priorities and methodologies must therefore be reimagined to align with local needs, while fostering dialogue between local and global communities to ensure equitable collaboration and knowledge exchange [58,62].

Taken together, these commitments do not constitute a fixed methodology but enabling conditions for a more just and pluralistic science—one that reclaims imagination as a vital faculty for collective epistemic transformation.

UNESCO’s vision of Open Science as a global good gestures toward equity, transparency, and collaboration, but without attention to the epistemological foundations of global health knowledge, these promises risk remaining rhetorical. The critiques we have traced, from feminist, decolonial, and Black feminist thought, show that exclusion is not an accident to be corrected but a constitutive feature of how science itself has been organized. This is why OS cannot be reduced to technical protocols or incremental reforms. A more just and pluralistic future for global health depends on reclaiming radical imagination: the capacity to envision science otherwise, through commitments that transform its purposes, boundaries, and relations of power. While this paper provides theoretical orientations for reimagining OS, the five commitments represent conceptual orientations rather than empirically tested interventions. Future work could explore how these insights translate into institutional practices in global health and examine their equity effects.
